# Mothers' and health workers' exposure to breastmilk substitutes promotions in Abidjan, Côte d'Ivoire

**DOI:** 10.1111/mcn.13230

**Published:** 2021-06-16

**Authors:** Jillian Emerson, Firmin Kouassi, Réné Oka Kouamé, Florence Neto Damey, Aita Sarr Cisse, Manisha Tharaney

**Affiliations:** ^1^ Alive & Thrive Initiative FHI Solutions Washington DC USA; ^2^ Alive & Thrive Initiative FHI Solutions Abidjan Côte d'Ivoire; ^3^ Laboratoire Anthropologie Physique et de Biomorphologie University Félix Houphouët Boigny Abidjan Côte d'Ivoire; ^4^ BEDSAH International Abidjan Côte d'Ivoire; ^5^ Programme National de Nutrition Ministère de la Santé, de l'Hygiene Publique et de la Couverture Maladie Universelle Abidjan Côte d'Ivoire

**Keywords:** Baby Friendly Hospital Initiative, breast milk substitutes, breastfeeding, child nutrition, infant formula, infant milk formula

## Abstract

Marketing of breastmilk substitutes (BMS) continues to undermine breastfeeding globally, and low income countries experiencing rapid economic growth are especially vulnerable as expanding BMS markets. The objective of the study was to understand the prevalence of exposure to BMS promotions among mothers of children 0–23 months, the frequency and type of contacts between BMS companies and health workers and the presence of educational/informational materials and branded equipment associated with such companies in health facilities in Abidjan using the World Health Organization's NetCode protocol. The methods included structured interviews with health workers and mothers and observations of equipment/materials in a sample of 42 facilities, 330 mothers and 129 health workers. Descriptive statistics were produced, and chi‐squared tests were used to assess differences by child age and facility type. Forty‐three per cent of mothers were advised to feed BMS products in the past 6 months, with a significantly higher percentage of mothers of older children (6–23 months) advised compared to infants 0–5 months. Two thirds (66%) of mothers had seen promotions outside of facilities. Among health workers, 63% were contacted by BMS companies, and only 8% were familiar with the International Code of Marketing of BMS. Differences were found between public/private facilities in the types of requests BMS companies made to health workers. Strong actions are needed in Côte d'Ivoire to prevent BMS promotion in the health system, including increasing health workers' knowledge of the International Code and national regulations, monitoring violations and reaching mothers and families to promote optimal breastfeeding practices.

**Key messages**

- BMS promotions at the community level and in the health system are widespread in Abidjan, Côte d'Ivoire.
- New mothers are commonly advised to feed BMS by health workers and family members and are exposed to BMS advertising through mass media.
- Strong actions are needed to prevent BMS promotions through the health system and community and increase health workers' knowledge of the Code and National BMS Decree; the implementation of a national Code monitoring and enforcement system is an important measure.

## 1 INTRODUCTION

Breastfeeding helps prevent more than 800,000 deaths of children under the age of five each year (Victora et al., 2016). In addition to preventing child morbidity and mortality and improving cognitive development, breastfeeding also reduces mothers' risk of breast and ovarian cancers and certain cardiovascular diseases (Neovita Study Group, 2016; Sankar et al., 2015; Victora et al., 2016). Exclusive breastfeeding, meaning giving infants only breastmilk and no other liquids or foods, for the first 6 months of life, is highly protective against morbidity and mortality, yet globally, only 41% of infants are exclusively breastfed from 0 to 5 months of age (Global Breastfeeding Collective, 2019; Sankar et al., 2015; World Health Organization et al., 2007). Many factors influence breastfeeding practices, but growing evidence suggests that marketing of breastmilk substitutes (BMS) has a strong influence, especially in growing low‐ and middle‐income country (LMIC) markets (Piwoz & Huffman, 2015). BMS companies began scaling up advertising and other promotions in LMICs in the 1970s, often employing marketing tactics such as giving gifts to health workers and deploying staff dressed as nurses to provide product donations directly to mothers (Brady, 2012). As the influence of such promotions spread, health providers and researchers noted significant increases in childhood illnesses and deaths linked to formula feeding (Muller, 1974). Not only were infants missing out on the immunological benefits of breastmilk, but often, mothers mixed products improperly or with contaminated water, exposing children to diarrhoea and other infections (Jelliffe, 1972). In recent years, BMS companies' marketing tactics have expanded to reach mothers through mothers' clubs, infant feeding advice hotlines, social media, gifts and promotional materials, as well as through more traditional channels such as the media and health care providers (Hastings, Angus, Eadie, & Hunt, 2020).

In 1981, the World Health Assembly (WHA) adopted the International Code of Marketing of Breastmilk Substitutes (the Code). Subsequent WHA resolutions provide details and guidance for its effective implementation to reduce mothers' and health workers' exposure to inappropriate BMS marketing. However, the International Code is not binding, and it is up to each member state to incorporate it into its national legislation and ensure its enforcement and monitoring. Significant gaps remain in adopting its various elements into national legislation (World Health Organization, 2020). The 2020 Status report on National Implementation of the International Code found that 136 of 194 World Health Organization (WHO) member states had enacted some Code provisions into national legislation. However, only 79 countries prohibit all forms of BMS promotion in health facilities, where health workers play a critical role in protecting and promoting breastfeeding. Pregnant women and new mothers are particularly vulnerable to BMS marketing tactics due to their reliance on health workers as a trusted source of infant feeding advice (Haroon, Das, Salam, Imdad, & Bhutta, 2013; Sanghvi, Jimerson, Hajeebhoy, Zewale, & Nguyen, 2013). Only 30 of 194 countries prohibit BMS companies from giving any type of gifts or incentives to health workers, according to the 2020 Status Report.

In West and Central Africa, seven of 24 countries have no legal measures in place, and only three countries have legal measures that are ‘substantially aligned’ with the Code—the Gambia, Ghana and Nigeria (World Health Organization, 2020). 1 Concurrently, West and Central Africa has the highest rate of under‐five mortality globally, with 97 children dying per 1,000 live births, and only 34% of infants under 6 months of age are exclusively breastfed, compared to 41% globally and 55% in Eastern and Southern Africa (UNICEF, 2019). Côte d'Ivoire, where Nestlé runs a large subsidiary producing infant foods, has one of the fastest‐growing global economies, expanding by 8% a year since 2012, with rising urbanization levels (The World Bank, 2019, 2020). Marketing and utilization of BMS is likely to be most common in large urban cities, where mothers often work outside the home, making exclusive breastfeeding more challenging (Barennes et al., 2012; World Health Organization & UNICEF, 2017). BMS consumption is correlated with greater household wealth in LMIC, perhaps due to its perception as modern and aspirational in some contexts (Neves et al., 2020). The prevalence of exclusive breastfeeding in Côte d'Ivoire rose from 12% to 23.5% between 2012 and 2016 (Ministere du Plan et du Developpement, UNICEF,,, & Insitut National de la Statistique, 2017), yet it remains far from the World Health Assembly Global Nutrition Target of 50% by 2025 (World Health Organization & UNICEF, 2014). Continued breastfeeding until age two is practised by only 36% of the wealthiest quintile of mothers in Côte d'Ivoire, compared with 76% of the poorest (UNICEF, 2019). BMS marketing may be an important factor undermining efforts to improve breastfeeding practices. Indeed, bottle feeding is six times higher in urban areas than in rural areas (Ministere du Plan et du Developpement et al., 2017). The government adopted National Decree 2013‐416 on 6 June 2013 which prohibits BMS promotion, regulates product labelling and stipulates sanctions for violations. However, little is known about the type and extent of BMS promotions occurring.

This study's objective was to evaluate the prevalence of exposure to BMS promotions among mothers and health workers in Abidjan, Côte d'Ivoire. The study assesses exposure to promotions among a facility‐based sample of mothers of children under 24 months of age, measures the prevalence of interactions between health professionals and company representatives and assesses the quantity and content of promotional material found in health facilities.

## 2 METHODS

### 2.1 Study design and context

The study was conducted using the NetCode Protocol for Periodic Assessments (World Health Organization & UNICEF, 2017). NetCode represents the Network for Global Monitoring and Support for Implementation of the International Code of Marketing of Breast‐milk Substitutes and subsequent relevant World Health Assembly Resolutions. In 2017, NetCode published two toolkits to support Code monitoring—one toolkit is designed to facilitate the development of an ongoing monitoring system, and the second toolkit provides guidance on conducting periodic assessments of Code violations. NetCode recommends conducting periodic assessments every 3 to 5 years to quantify the level of compliance with the International Code and national legislation so that programme managers and policymakers may identify and address areas where there are gaps in enforcement of the law.

There are three modules in the NetCode Protocol for Periodic Assessments. The Retail and Label Module assesses the extent of promotions in BMS points of sale, such as pharmacies, supermarkets and online, and the level of adherence of product label to the Code. The Mass Media Module measures the prevalence and type of BMS promotion in television and internet. Due to limited resources, the present study included only the Mothers and Health Facilities Module, which assesses the prevalence of exposure to BMS marketing of mothers of children under 24 months of age attending health facilities, the prevalence and type of interactions between health professionals and BMS company representatives and the quantity and content of promotional materials found at public and private health facilities.

With regard to the scope of the products covered, the Code stipulates that BMS may include ‘infant formula; other milk products, foods and beverages, including bottlefed complementary foods, when marketed or otherwise represented to be suitable, with or without modification, for use as a partial or total replacement of breast milk’, in addition to feeding bottles and teats (World Health Organization, 1981). WHA resolution 69.9 provides guidance for all commercially produced foods that are marketed as being suitable for infants and young children 6–36 months of age and further clarifies that BMS may include any milks that are specifically marketed to feed infants and young children up to 3 years of age (World Health Organization, 2016). The scope of the National Decree is similar to that of the Code but does not take into account the recommendations of Resolution 69.9. It prohibits the marketing of BMS or other milk products, foods or drinks, including complementary foods, when they are marketed to replace breastmilk, as well as bottles, teats and similar products (Decret 2013‐416 du 6 juin 2013 portant reglementation de la commercialisation des substituts du lait maternel, 2013). It is important to note the study assessed the type of feeding advice given by health workers to mothers, as well as the prevalence of interaction between BMS company representatives, which do not represent violations of the Code or the National Decree but may negatively impact breastfeeding practices. The Code and the Decree do not prohibit the dissemination of information by BMS companies to health care professionals as long as the materials are restricted to scientific and factual matters and otherwise comply with the Code. In addition, Article 6.2 of the Code states that no facility should be used to promote infant formula or other products within the scope of the Code. Neither the Code nor the Decree address health worker advice to mothers; however, Article 7.1 of the Code states that health workers should encourage and protect breastfeeding.

The study was conducted in Abidjan, the economic capital and largest city in Côte d'Ivoire. The study was a cross‐sectional survey conducted in October 2019. Methods included structured interviews of mothers and health workers and observation of equipment and materials in health facilities to ascertain whether they were produced or donated by BMS companies. Ethical approval for the study was received from the National Ethics Committee for Life Sciences and Health (CNESVS) and from the Institutional Review Board of FHI 360. The Ministry of Health and Public Hygiene (MSHP) through the National Nutrition Program (PNN) of the government of Côte d'Ivoire authorized this study. Informed consent was obtained from all study participants.

### 2.2 Participants and sampling

The sampling strategy and target sample sizes were chosen following the guidance provided in the NetCode protocol.

#### 2.2.1 Health facilities

The first stage of sampling involved drawing a sample of 33 primary health facilities and 10 secondary level maternities, as stipulated in the NetCode protocol. The Ministry of Health's Health Management Information System (DHIS2) was used to generate a list of primary and maternity facilities in Abidjan and their utilization rates for child health services (vaccination, growth monitoring and/or nutrition counselling services). Any facility offering such services was eligible for selection. Although the use of public health services has increased in recent years, it is estimated that one quarter of health services are provided by private providers (Oxford Business Group, 2020). To ensure sufficient representation of private facilities, we stratified the sampling frame by facility type. Overall, there were 110 public facilities and 35 private facilities in the sampling frame. We selected 25 public facilities and eight private facilities randomly using probability proportional to size (PPS) sampling (based on utilization rates for child health services), with one facility sampled twice resulting in a final sample of 32 primary facilities. Maternities were also stratified by the type of facility (public or private), and then, six public and four private facilities were selected, which had the highest number of deliveries of each type.

#### 2.2.2 Mothers

At each health facility, mothers of children under 6 months of age and mothers of children 6–23 months of age receiving child health services and who provided informed consent were eligible for participation. Mothers were selected using a convenience sampling strategy, wherein eligible mothers were sampled consecutively until five; mothers of children under 6 months of age and five mothers of children 6–23 months of age were interviewed in each facility. A total of 330 mothers participated. Because one facility was sampled twice, two groups of mothers from that facility participated. No mothers attending the maternity facilities participated in the study as per the NetCode protocol.

#### 2.2.3 Health workers

Three health professionals were interviewed in each facility (six from the facility that was sampled twice) and in each maternity. The director, doctor, nurse, midwife and/or health worker assigned to the paediatrics, obstetrics and gynaecology departments were eligible and were invited to participate. The first three health workers who met these criteria and were available for the interview participated, totalling 129 from the 43 primary facilities and maternities sampled.

### 2.3 Data collection

Prior to data collection, study supervisors and enumerators participated in a 4‐day training on research ethics and study methods and to become more familiar with the data collection tools. The questionnaire was also pilot tested in one health centre in Abidjan. Data collection took place in October 2019. The research team made appointments with the lead health professional in each of the facilities selected, conducted interviews with him or her at the designated time and then requested introductions to other health workers in the facility who met the eligibility criteria described previously. When a health worker declined to participate, the data collection team approached another health worker who met eligibility criteria. The study team then approached mothers of children 0–5 months of age and 6–23 months of age while they were waiting for well‐child health services (growth monitoring and immunization) and asked them if they were willing to participate. Mothers were interviewed in a semi‐private area away from the other mothers waiting to receive services.

Data were collected electronically using the Open Data Kit (ODK) application on smartphones (Hartung et al., 2010). Three types of tools were used: a questionnaire for mothers of children 0–23 months, a questionnaire for health workers and an observation guide to assess promotional and information/education material in the health facilities. The mothers' questionnaire first asked mothers whether they had been advised to feed any milk product other than breastmilk in the past 6 months. If their response was ‘yes’, they were asked who made the recommendation, what type of product it was (infant formula, follow up/on formula, growing up milk, baby milk for an unspecified age range, milk not targeted for babies or a combination of milk product categories) and what brand it was. Mothers had the opportunity to list multiple products if they were advised to use them more than once in the last 6 months. Mothers were also asked if anyone advised them to feed their child any other food or drinks when the child was (or if the child is currently) under 6 months of age and the type of product recommended. Response options included complementary foods or liquid, a combination of product categories or not specified. Finally, mothers were asked if they had heard or seen any promotions at the health facility such as posters, flyers and videos or were exposed to promotional messages for any milk products, bottles or teats for children under 3 years of age through the media or in the community in the past 6 months.

The health worker questionnaire assessed whether health workers had been contacted by any personnel from companies that sell baby foods, bottles or teats in the past 6 months, and if so, how the contact was made, the reason that the company made contact and the frequency by which the company made contact. If multiple companies contacted health workers, the same information was collected for each. Health workers were also asked if they had attended any professional conferences or scientific meetings in the past 2 years sponsored by such companies, their familiarity with the International Code and national laws and regulations on BMS marketing and whether they had ever received training on infant and young child feeding (IYCF), the International Code or any national BMS marketing measures.

Any promotional and educational/information materials, as well as equipment bearing the logo of BMS companies found in the health facility, and the type of product promoted (infant formula, follow up/on formula, growing up milk, any other milk for children 0–36 months, any other food or liquid for infants, commercial complementary foods, feeding bottles or teats or not a specific product) were noted using an observation guide.

### 2.4 Analysis

Data were exported from the online questionnaires and were reviewed for any outlying or missing values and checked for consistency between questionnaires. Data from the observations were entered into an Excel file. Descriptive analyses were conducted using STATA, and frequencies were evaluated for each variable. Chi‐squared tests were used to check for any statistically significant differences in BMS promotion exposure between mothers of children 0–5 and 6–23 months, as well as between mothers attending public compared to private facilities. We also conducted chi‐squared tests to assess differences in health workers' exposure to BMS contacts by type of facility (public compared to private).

## 3 RESULTS

### 3.1 Participant characteristics

Participant characteristics can be found in Table 1. There were 165 mothers with children under 6 months and 165 with children 6–23 months who participated. Almost all (98.8%) had only one child under two. About one third of mothers (32.1%) had no formal education, 28.2% had some primary educationand 29.7% had at least some secondary education. Relatively fewer (10%) had any post‐secondary education. There were no differences in participant characteristics among mothers attending private compared to public facilities. Among health workers surveyed (*n* = 129), many were midwives (41.9%). Other respondents included nurses (14%), doctors (11.6%), department heads (8.5%), health aids (10.9%) and other positions (13.2%). About half had 1 to 5 years of experience in their role.

**TABLE 1:** Participant characteristics

	*N* (%)
**Mothers (*N* = 330)**	
Child age <6 months	165 (50)
Child age 6–23 months	165 (50)
Has only one child <2 years of age	326 (98.8)
Education	
No formal education	106 (32.1)
At least some primary	93 (28.2)
At least some secondary	98 (29.7)
Some post‐secondary	33 (10)
**Health workers (*n* = 129)**	
Type of position	
Midwife	54 (41.9)
Doctor	15 (11.6)
Nurse	18 (14.0)
Department head	11 (8.5)
Health aid	14 (10.9)
Other	17 (13.2)
Years of experience	
Less than a year	13 (10.1)
1 to 5 years	64 (49.6)
6–10 years	29 (22.5)
11–15 years	11 (8.5)
16+ years	12 (9.3)

### 3.2 Mothers' exposure to BMS promotions

Data on mothers' exposure to BMS promotions both inside and outside of health facilities are displayed in Table 2. Among the mothers with infants under 6 months of age (*n* = 165), a time when exclusive breastfeeding is recommended by the WHO, more than half (57.6%) were advised to start feeding their infants food or drinks other than breastmilk. Almost half (43.3%) of all mothers in the sample were advised to feed BMS products in the past 6 months. There were significant differences between children's age groups, with 56.8% of mothers of children 6–23 months being advised to feed BMS, compared to 29.7% of mothers of children 0–5 months (*p* < 0.05). Among all the mothers, about 27% received this advice two or more times, resulting in a total of 193 instances in which mothers received advice about feeding BMS products in the past 6 months, and no significant differences in advice frequency were found between child age groups. The advice mothers received did not differ between mothers attending public and private facilities either.

**TABLE 2:** Mothers' exposure to BMS promotions inside and outside health facilities

	*N* (%)		
1. Prevalence of mothers of *infants <6 months* advised to start feeding infant *any other food or drink* (*n* = 165)	95 (57.6)		
2. Prevalence of mothers advised to feed *milk products other than breastmilk* in the past 6 months, by age of child (*n* = 330)	**0–5 months**	**6–23 months**	**0–23 months combined**
	**49 (29.7)**	**94 (56.8)**	**143 (43.4)**
a. Number of times mothers were advised to feed milk products			
Once	38 (77.6)	66 (70.2)	104 (72.7)
Two or more times	11 (22.5)	28 (29.8)	89 (27.3)
b. Among the 193 times mothers were advised to feed milk products, type of product recommended			
Infant formula	**60 (95.2)**	**68 (52.3)**	**128 (66.3)**
Follow‐up/on formula	**2 (3.2)**	**46 (35.4)**	**48 (24.9)**
Milk product (age range not specified/unknown)	**0**	**6 (4.6)**	**6 (3.1)**
Growing up milk	**0**	**3 (2.3)**	**3 (1.6)**
A combination of milk product categories	**0**	**5 (3.9)**	**5 (2.6)**
Product not specified	**1 (1.6)**	**2 (1.5)**	**3 (1.6)**
c. Company whose product(s) was/were recommended (*n* = 193)			
France Lait	8 (10.8)	14 (10.8)	22 (11.4)
Nestlé	14 (22.2)	24 (18.5)	38 (19.7)
Danone	21 (33.30)	51 (39.2)	72 (37.3)
Unknown	15 (23.8)	19 (14.6)	34 (17.6)
Other	5 (7.9)	22 (16.9)	27 (14.0)
d. Who recommended product(s) (*n* = 193)			
Midwife	10 (16.4)	26 (20.2)	36 (19.0)
Paediatrician	11 (18.0)	15 (11.6)	26 (13.7)
Other health professional	5 (8.2)	18 (14.0)	23 (12.1)
Parent/husband/friend	20 (32.8)	38 (29.5)	58 (30.5)
Store or pharmacy staff	11 (18.0)	21 (16.3)	32 (16.8)
BMS company representative	1 (1.6)	4 (3.1)	5 (2.6)
Do not remember	1 (1.6)	1 (0.8)	2 (1.1)
Other	2 (3.3)	6 (4.7)	8 (4.2)
3**.** Promotional messages: in the past 6 months, prevalence of mothers who had:			
a. Heard or seen any poster, flyer/brochure, video, or other promotional materials *at this health facility*	8 (4.9)	15 (9.1)	23 (7.0)
b. Heard or seen promotional message from TV, radio, magazines, shop/pharmacies, billboards, social media, internet, or community event	105 (63.6)	114 (69.1)	219 (66.4)
1. Type of exposure among the 347 instances of mothers' exposure through media and community			
Television	129 (82.7)	149 (78.0)	278 (80.1)
Advertising in stores/pharmacies	13 (8.3)	28 (14.7)	41 (11.8)
Billboard	9 (5.8)	9 (4.7)	18 (5.2)
Internet/social media	1 (0.64)	3(1.6)	5 (1.4)
Radio	0	2 (1.1)	2 (0.6)
Other	4 (2.5)	0	4 (1.2
c. Received free samples of any baby milk products	6 (3.6)	14 (8.5)	20 (6.1)
d. Received a coupon for any baby milk products or feeding bottles and teats for children less than 3 years old	1 (0.6)	0	1 (0.3)

*Note:* Results in bold indicate chi‐squared test differences with *p* < 0.05.

Within these 193 instances, 66.3% of the products recommended were infant formulas, and about 25% were follow on formulas. There were significant differences between the types of BMS products recommended by age group, with a much higher percentage of mothers of children 0–5 months being recommended infant formula (95.2%) compared to mothers of children 6–23 months (52.3%), and a higher percentage of mothers of children 6–23 months recommended follow‐up formula (35.2%), versus 3.2% of mothers of infants 0–5 months (*p* < 0.05). Some of the mothers with older children were also recommended growing up milk or various combinations of milk product categories. There were no differences in types of products recommended by mothers attending public compared to private facilities. Francelait, Danone and Nestlé were commonly cited producers of the recommended products, with no variation between facility type or child age. Sources of recommendations for BMS products were health centre staff including midwives (19.0%) and paediatricians (13.7%), as well as close friends and family members (30.5%) and store/pharmacy staff (16.8%), and no differences were found between public and private facilities or child age. In the past 6 months, very few (7%) mothers had reported seeing any BMS product promotional material in the health facility. However, about two thirds (66.4%) had seen or heard BMS promotions through the media and community, including TV, radio, magazines, shop/pharmacy ads, billboards, social media, internet or community events. Among these 219 mothers reporting any exposure to promotions through the media and community, there were a total of 347 instances of exposure, because some mothers reported multiple instances and channels of exposure. The channels by which mothers were exposed included television (80.1% of instances) and stores/pharmacies (11.8%), with very few mothers reporting exposure through radio, internet and social media.

About 6% of mothers had received a free sample of a BMS product, only one mother had received a coupon and no mothers had received any gifts from BMS companies. Among the mothers receiving samples, they most often reported receiving multiple samples. The sources included BMS company representatives, health professionals and retail outlets, as well as partners, parents and friends. Although the data are not displayed in Table 2, the study also assessed whether mothers had participated in any online or in‐person social groups or events for mothers and, if so, if they were sponsored by BMS companies. Only 6% of mothers participated in any online or in‐person social groups. Although 41% of mothers reported participating in a parenting or IYCF class, only six, or 4%, of these mothers said a BMS company sponsored the class. No variations were found between child age groups.

### 3.3 Health facilities and staff

Results from interviews with health facility staff are reported in Table 3. More than half of staff (62.8%) had been contacted by a representative of a BMS company in the past 6 months, often by multiple companies. No statistically significant differences were found in the number of contacts between private and public facilities, or cadre of staff. Of the 81 facility staff who had been contacted, a total of 174 contacts were made, with a large proportion of these contacts by representatives of Danone (28.2%) and Nestlé (21.8%). There were 65 health workers who were contacted by Danone or Nestlé at least once, which represents 80.2% of all health workers who had any contacts with BMS companies.

**TABLE 3:** Contacts between companies and health facility staff (*N* = 129)

	Public	Private	Total
1. Prevalence of health workers who have been contacted at least once by any personnel from companies who sell baby foods, bottles, or teats in the past 6 months	70 (66.7)	11 (45.8)	81 (62.8)
a) By only one company	24 (34.3)	4 (36.4)	28 (34.6)
b) By two companies	26 (37.1)	1 (9.1)	27 (33.3)
c) By three or more companies	20 (28.6)	6 (54.6)	26 (32.1)
2. Among the 81 health workers contacted by companies, the prevalence of contacts (*n* = 175):			
a) By company			
Danone	42 (28.8)	7 (25)	49 (28.2)
Nestlé	32 (21.9)	6 (21.4)	38 (21.8)
Pharmalys Laboratory	7 (4.8)	2 (7.1)	9 (5.2)
France Lait	12 (8.2)	3 (10.7)	15 (8.6)
Primalac	11 (7.5)	3 (10.7)	14 (8.1)
Other	30 (20.6)	4 (14.3)	34 (19.5)
Do not know	12 (8.2)	3 (10.7)	15 (8.6)
b) By means of contact			
Telephone	7 (4.8)	0	7 (4.0)
Email	2 (1.4)	0	2 (1.2)
Onsite visit	128 (86.3)	27 (96.4)	153 (87.9)
Mail	1 (0.7)	0	1 (0.6)
Other/missing	10 (6.8)	1 (3.6)	11 (6.3)
c) In which companies reported the intent to distribute *to mothers*:			
Promotional materials for certain products	55 (37.7)	14 (50.0)	69 (39.6)
Other materials for education and information	53 (36.3)	11 (39.3)	64 (36.8)
Samples or other products for child feeding	39 (26.7)	8 (28.6)	47 (27.0)
Gifts	25 (17.1)	5 (17.9)	30 (17.2)
Coupons	1 (0.7)	0	1 (0.6)
d) In which company representatives reported the intent to distribute *to the health facility*:			
Promotional material	34 (23.3)	10 (35.7)	44 (25.3)
Information/educational material	76 (52.1)	23 (82.1)	100 (57.5)
Gifts	31 (21.2)	7 (25.0)	38 (21.8)
Displays	11 (7.5)	1 (3.6)	12 (6.9)
e) In which companies' representatives requested or offered to:			
Establish direct contact with mothers	**34 (23.3)**	**15 (53.6)**	**49 (28.2)**
Establish direct contact with staff	118 (80.8)	21 (75.0)	139 (79.0)
Provide free supplies of BMS and other child feeding products	37 (25.3)	9 (32.1)	46 (26.4)
Provide donations of equipment	**10 (6.9)**	**13 (46.4)**	**23 (13.2)**
Sponsor workshops for health facility staff	**25 (17.1)**	**10 (35.7)**	**36 (20.7)**
Provide support for health facility staff to attend workshops	**13 (46.4)**	**40 (27.4)**	**54 (31.0)**
Provide other offers	2 (7.1)	24 (16.4)	25 (14.4)
3. Among health facility staff who attended a conference/meeting in the past 2 years (*n* = 70), prevalence of conference/meetings attended sponsored by a company	15 (26.3)	4 (30.8)	19 (27.1)
4. Prevalence of health facility staff who reported:			
a) Being familiar with International BMS Code	11 (10.5)	0	11 (8.5)
b) Being familiar with national laws or regulations on the marketing of BMS	13 (12.4)	2 (8.3)	15 (11.6)
c) Ever receiving training on breastfeeding or IYCF	69 (65.7)	13 (54.2)	82 (63.6)
d) Ever receiving training on the International BMS Code	1 (1.0)	0	1 (0.7)
e) Ever receiving training on national laws or regulations on BMS marketing	1 (1.0)	0	1 (0.7)

*Note:* Results in bold indicate chi‐squared test differences with *p* < 0.05.

In‐person visits to the facility were the most prevalent means (87.9%) of contacting health workers. The goals of the contacts by BMS companies were to provide the health facilities with promotional material (39.6% of contacts) for BMS products, educational and informational materials (36.8%), BMS samples and child feeding products (27.0%) or gifts (17.2%) targeted at mothers. They also offered to provide the health facility with promotional material (25.3%), informational and educational material (57.5%) and gifts (21.8%) and displays (6.9%) targeted at health workers. In 28.2% of contacts, BMS companies requested to establish direct contact with mothers, and in 79%, they requested to make direct contacts with staff. Other offers included providing free supplies of BMS and other feeding products (26.4%), providing donations of equipment (13.2%), sponsor workshops for health facility staff (20.7%) and provide support for health facility staff to attend workshops (31.0%). There were significant differences between public and private facilities in some of the requests and offers that BMS companies made to health workers. A higher prevalence of requests to distribute promotional material was found in private compared to public facilities (53.6% compared with 23.3%), as well as offers to provide donations of equipment (46.4% compared with 6.9%), requests to establish direct contacts with mothers (53.6% compared with 23.3%) and sponsor in‐facility workshops for health facility staff (35.7% compared with 17.1%; *p* < 0.05). A higher prevalence of offers to provide support for staff to attend external workshops was found in public (46.4%) compared to private facilities (27.4%; *p* < 0.05). No differences were found between public and private facilities in terms of the prevalence of requests to establish direct contact with staff (79%), provide free BMS and other child feeding products (26.4%) and provide any other offers (14.4%).

Among the 70 health facility staff who had attended any type of conference or meeting in the last 2 years, about one third (27.1%) reported that the meeting was sponsored by a BMS company. Only 8.5% of health facility staff respondents were familiar with the International BMS Code, and just a few more (11.6%) were familiar with the National BMS Decree. Although 63.6% of respondents had received breastfeeding or infant and young child feeding training, only one reported ever receiving training on the International Code or the National BMS Decree. No differences in conference attendance or Code‐related training and knowledge were found between health workers in public versus private facilities.

The number and type of promotional and educational material found in health facilities are shown in Table 4. We found a total of 66 BMS‐branded materials in 26 health facilities. They ranged from cups (9.1%) to brochures/posters (15.2%), notebooks (7.6%), equipment such as height boards and stethoscopes (12.1%) and child clothing and feeding accessories (9.1%). Most of the materials (95.5%) were found in primary health facilities, rather than maternities. The general public was the target audience for about 64% of materials found, and health workers were the target for 27%. About one third of the materials had branding that did not focus on a particular product, but follow up/on formula (21.2%), growing up milk (15.2%) and complementary food products (15.2%) were specific types of products mentioned the most. About one quarter of products were branded by Pharmalys Laboratoire, whereas France Lait (16.7%), Nestlé (15.2%), Bledina (10.6%) and PKL (10.6%) were also common brands across these materials.

**TABLE 4:** Equipment, promotional and education/informational materials bearing BMS company logos (*n* = 66)

a) Material found at	
Primary health facility	63 (95.5)
Maternity facility	3 (4.6)
b) Target audience	
General public	42 (63.6)
Health workers	18 (27.3)
Other	6 (9.1)
c) Type of product being advertised	
Infant formula	2 (3.0)
Follow up/on formula	14 (21.2)
Growing up milk	10 (15.2)
Other milk for infants	5 (7.6)
Complementary food product	10 (15.2)
Feeding bottles or teats	2 (3.0)
No specific product (e.g. company logo only)	23 (34.9)
d) BMS company	
Danone	3 (4.5)
Nestle	10 (15.2)
Pharmalys Laboratoire	19 (28.8)
Bledina	7 (10.6)
PKL	7 (10.6)
Francelait	11 (16.7)
Other/unknown	9 (13.6)
e) Type of promotional material	
Cups	6 (9.1)
Brochures and posters	10 (15.2)
Calendars	14 (21.2)
Notebooks	5 (7.6)
Product samples	3 (4.5)
Clothes and feeding accessories for children	6 (9.1)
Equipment such as scales, smocks and stethoscopes	8 (12.1)
Other materials	14 (21.2)

## 4 DISCUSSION

Our study found that mothers of young children in Abidjan frequently received advice to feed BMS products to their infants and young children, including more than half of mothers of infants under 6 months of age. They often received this advice from health professionals, including midwives and paediatricians as well as members of their family and community and in pharmacies or stores. Mothers of children 6–23 months of age had a significantly higher prevalence of being advised to feed BMS than mothers with children under 6 months, but no differences were found in advice received between mothers attending public compared with private facilities. About two thirds of mothers had been exposed to BMS marketing outside of the health facility, through mass media and other advertising. More than half of health facility staff interviewed were contacted by BMS companies in the past 6 months, many of whom had been contacted multiple times, most often through in‐person visits by company representatives. BMS companies were significantly more likely to target private facilities to establish direct contact with mothers, provide donations of equipment and sponsor on‐site trainings for staff, whereas they more often offered support for public health facility staff to attend external workshops. Very few health workers were familiar with either the International BMS Code or the National BMS Decree. Despite relatively few mothers reporting seeing IYCF product‐ branded materials at health facilities, a total of 66 materials were found, ranging from height boards, to informational pamphlets, to smocks designed for health workers to wear.

The prevalence of BMS promotion exposure reported by mothers and health workers that we found in Abidjan was much higher than those reported by other studies in sub‐Saharan Africa. A situational analysis conducted in Dakar, Senegal, revealed a lower level of BMS promotions in the health system, with only about one fifth of mothers reporting that a health worker had recommended BMS products to them or observed branding for either BMS or commercially produced complementary foods (Feeley et al., 2016). About 40% of mothers were exposed to any promotions outside the health facility. In Tanzania, the prevalence of exposure was quite low, with only 9% of mothers receiving recommendations from a health professional to feed BMS, and 10% were exposed to promotions inside or outside of the facility (Vitta et al., 2016). The results were comparable to several studies from Asia. In Nepal, the Assessment and Research on Child Feeding (ARCH) project found that almost half of the mothers they surveyed were recommended BMS products by health workers (Pries, Huffman, Adhikary, et al., 2016). Few mothers reported observing promotional materials in the facilities or receiving samples. In Cambodia, almost one fifth of mothers were recommended BMS products by a health professional, but 86% observed BMS promotions outside of health facilities (Pries, Huffman, Mengkheang, et al., 2016). In Latin America, one study in Mexico found that more than half of mothers surveyed had been advised to feed BMS products to their children, often by health professionals, and 80% of them had been exposed to violations outside of the health facility (Instituto Nacional de Salud Pública, Universidad Iberoamericana, & UNICEF México, 2020).

Although Côte d'Ivoire has a national Decree to regulate BMS marketing, to date, its provisions have not been enforced. An important step to reduce BMS promotions in health facilities would be to train health workers on the International Code and National Decree and their responsibilities therein, given that virtually none participating in this study had even heard of the Code. Health worker training on the Code has been incorporated in plans to scale up the Baby‐Friendly Hospital Initiative (BFHI), and the Code and the BHFI's Ten Steps to Successful Breastfeeding should be integrated into ongoing capacity‐building efforts for health workers and managers across the health system (UNICEF & World Health Organization, 2018). The government of Côte d'Ivoire may consider reviewing the contents of the decree to determine if it adequately protects mothers and babies from inappropriate marketing.

For example, stricter provisions may be necessary to control the contacts between marketing personnel and health workers. Additionally, the implementation of an ongoing monitoring system to quickly identify violations and the imposition of sanctions is needed to strengthen the application of the Decree. Although there are few examples of successful monitoring systems, a pilot in Cambodia found it feasible to monitor violations using existing government platforms (Hou et al., 2019).

Although Nestlé and Danone were frequent violators, there were many smaller companies who were also active in engaging in promotional activities within the health sector. Careful attention should be paid to their growth and influence, as their promotional tactics may be less noticeable to authorities. The study found that BMS companies reached out to private facilities more often requesting to make direct contacts with mothers, provide donations of equipment and sponsor workshops for staff compared to public facilities, so it will be important to ensure private facilities are included in any future monitoring and enforcement efforts and that health system managers are aware that such actions represent violations. Mothers of children 6–23 months of age were most often advised to feed BMS products, which may indicate that a greater focus should be on promoting continued breastfeeding to 2 years and beyond, as well as age‐appropriate complementary feeding. Many children's growth falters in this transitional period as more calories are required to supplement breastmilk (Shrimpton et al., 2001), so mothers may be turning to BMS to assuage fears about their children's nutritional status. It is also important to note that almost one third of mothers were advised to give BMS products by friends or family members. Thus, efforts to reach these influential groups at the household and community level are needed to sensitize them to the risks of using BMS products and the benefits of practising optimal IYCF behaviours.

One of our study's limitations was that we only implemented the ‘Mothers and Health Facilities’ module of the NetCode protocol and did not assess violations in retail (e.g., stores and pharmacies) and the media that are also part of NetCode. We are, therefore, not able to present a complete picture of the extent of BMS promotions in Abidjan. Additionally, because the study took place in Abidjan and only among mothers seeking services at health facilities, it is not representatives of mothers' exposure to BMS promotions in all of Abidjan or Côte d'Ivoire. However, when resources are not available to conduct a nationally representative or multi‐site assessment, NetCode recommends conducting the assessment in a country's economic capital only, where BMS marketing tends to be the most prevalent. The study's main strength is that it employs the NetCode protocol, a standardized tool developed by International BMS Code experts specifically to identify Code violations. The results will be comparable with studies in other geographic areas that use the same methodology. Another strength was the mixed‐methods approach that combined semi‐quantitative interviews with observations, allowing the research team to triangulate findings and obtain a full picture of the type and extent of BMS promotions.

## 5 CONCLUSION

Côte d'Ivoire is positioned to become a middle‐income country in the next 15 years (The World Bank, 2019); however, the pervasive promotion of BMS products and undermining of breastfeeding may contribute to increased risk of avoidable illnesses, death and poor physical and cognitive development among the next generation of Ivoirian children. Increasing mothers' adoption of optimal breastfeeding practices that save lives and provide infants with the healthiest start in life, including early initiation of breastfeeding and exclusive breastfeeding for the first 6 months of life, requires strong actions from the policy down to the household level. Strengthening policies and systems to prevent the promotion of BMS products is a critical step in creating an enabling environment for mothers to breastfeed. Côte d'Ivoire and other LMIC that are rapidly developing are especially vulnerable to BMS companies' marketing efforts and often lack strong systems in which product marketing can be regulated and violations monitored. As BMS companies continually adapt the channels in which they market products to have increasingly high reach, governments and health and nutrition sector partners will need to be increasingly vigilant in detecting International Code violations and protecting mothers and infants.

## CONFLICTS OF INTEREST

The authors declare no conflict of interest.

## CONTRIBUTIONS

All authors contributed to the design of the study and the interpretation of the results and critically reviewed the manuscript. JE drafted the manuscript and conducted final analyses. FK conducted the research and preliminary analyses. FD and RO oversaw field data collection.

## Data Availability

The data that support the findings of this study are available on request from the corresponding author. The data are not publicly available due to privacy or ethical restrictions.

## Footnotes

^1^
Sao Tome & Principe and Mauritania adopted laws based on the Code after the publication of the 2020 Status Report.
